# Beyond the Secretory Pathway: New Insights Into Protein Release

**DOI:** 10.1111/tra.70022

**Published:** 2025-11-06

**Authors:** Ruey‐Hwa Chen, Antonio J. Costa‐Filho, Jayanta Debnath, Thierry Galli, Liang Ge, Deborah Goberdhan, Wei Guo, Kangmin He, Ralf Jacob, Tiebang Kang, Min Goo Lee, Lian Li, Fabio Lolicato, Jun Lu, Vivek Malhotra, Walter Nickel, Vassiliki Nikoletopoulou, Stacey K. Ogden, Georgia Maria Sagia, Feng Shao, Anbing Shi, Clotilde Thery, Christel Vérollet, Julien Villeneuve, Frederik Verweij, Yanzhuang Wang, Juan Wang, Shenjie Wu, Yihong Ye, Hang Yin, Li Yu, Min Zhang, Ying Zhang, Xin Zhou, Chiara Zurzolo

**Affiliations:** ^1^ Academia Sinica Taipei Taiwan; ^2^ University of São Paulo São Paulo Brazil; ^3^ University of California San Francisco San Francisco California USA; ^4^ Université Paris Cité Institute of Psychiatry and Neuroscience of Paris, INSERM Paris France; ^5^ Tsinghua University Beijing China; ^6^ University of Oxford Oxford UK; ^7^ University of Pennsylvania Philadelphia Pennsylvania USA; ^8^ Institute of Genetics and Developmental Biology, Chinese Academy of Sciences University of Chinese Academy of Sciences Beijing China; ^9^ Philipps University of Marburg Marburg Germany; ^10^ Sun Yat‐Sen University of Cancer Center Guangzhou China; ^11^ Yonsei University Seoul South Korea; ^12^ Emory University School of Medicine Atalanta Georgia USA; ^13^ Heidelberg University Biochemistry Center Heidelberg USA; ^14^ Yale University New Haven Connecticut USA; ^15^ Centre for Genomic Regulation Barcelona Spain; ^16^ Universite de Lausanne Lausanne Switzerland; ^17^ St. Jude Children's Research Hospital Memphis Tennessee USA; ^18^ National and Kapodistrian University of Athens Athens Greece; ^19^ National Institute of Biological Sciences Beijing China; ^20^ Huazhong University of Science and Technology Wuhan China; ^21^ Institut Curie Paris France; ^22^ CNRS‐University of Toulouse Toulouse France; ^23^ Institute of Functional Genomics (IGF) University of Montpellier Montpellier France; ^24^ University Utrecht Utrecht the Netherlands; ^25^ Shenzhen Bay Laboratory Shenzhen China; ^26^ Beijing University of Technology Beijing China; ^27^ University of California Berkeley California USA; ^28^ National Institutes of Health Bethesda Maryland USA; ^29^ Harbin Medical University Harbin China; ^30^ Pasteur Institute Paris France

**Keywords:** autophagy, CUPS, extracellular vesicle, lysosome, stress adaptation, unconventional protein secretion

## Abstract

In eukaryotes, protein secretion plays essential roles in intercellular communications and extracellular niche‐building. Protein secretion generally requires a signal sequence that targets cargos to the canonical secretory pathway consisting of the endoplasmic reticulum (ER), the Golgi apparatus, plasma membrane, and vesicles moving between these compartments. However, cytoplasmic proteins lacking signal sequences (e.g., IL1β, Acb1, FGF2) have been detected, and many have defined functions in the extracellular space, suggesting unconventional protein secretion (UcPS) via alternative pathways. In recent years, scientists have uncovered many new UcPS paradigms, reporting a plethora of mechanisms that collectively form a new field. The inaugural Cold Spring Harbor Asia (CSHA) conference on “Molecular Mechanisms and Physiology of Unconventional Secretion*”* is the first meeting to bring these researchers together, providing a collegial platform for information sharing at this exciting frontier of cell biology research.

## Graphical abstract

While proteins bearing N‐terminal signal or leader sequences are secreted via the ER‐Golgi‐plasma membrane axis of the conventional secretion pathway, many cytosolic proteins lacking signaling sequence can be secreted via poorly defined mechanisms termed Unconventional Protein Secretion (UcPS).

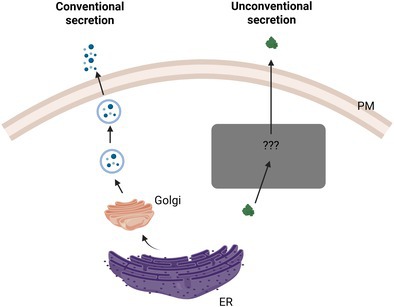

## 1 Introduction

In March 2025, Cold Spring Harbor Asia hosted its inaugural conference of the year entitled “Molecular Mechanisms and Physiology of Unconventional Secretion.” Organized by a panel of internationally renowned scholars including Drs. Yihong Ye (National Institutes of Health), Wei Guo (University of Pennsylvania), Walter Nickel (Heidelberg University), Min Goo Lee (Yonsei University), and Min Zhang (Tsinghua University), the conference convened more than 100 experts, scholars, and trainees. The four‐and‐a‐half‐day conference included two keynote speeches, 23 invited talks, 24 selected short talks, and two poster sessions. These presentations reported various new findings on the mechanisms of unconventional protein secretion, implicating almost all subcellular organelles including the endoplasmic reticulum (ER), the Golgi apparatus, endosomes, lysosomes, and autophagosomes. This conference is the only one to date centered around unconventional protein secretion, a relatively unexplored cell biology direction that just starts to gain attention in recent years.

Although the field of unconventional protein secretion is relatively young, its roots trace back to the foundational work of Nobel laureates George Palade and Günter Blobel, whose discoveries on vesicle‐mediated trafficking and signal sequence–based protein targeting defined the canonical secretory pathway via the ER–Golgi–plasma membrane axis [1, 2]. Soon after, it became clear that certain proteins lacking signal sequences could also be secreted [3], implying the existence of an alternative pathway outside the canonical paradigm. This process was termed unconventional protein secretion (UPS), but to avoid confusion with the ubiquitin–proteasome system, we refer to it as UcPS.

**FIGURE 1:**
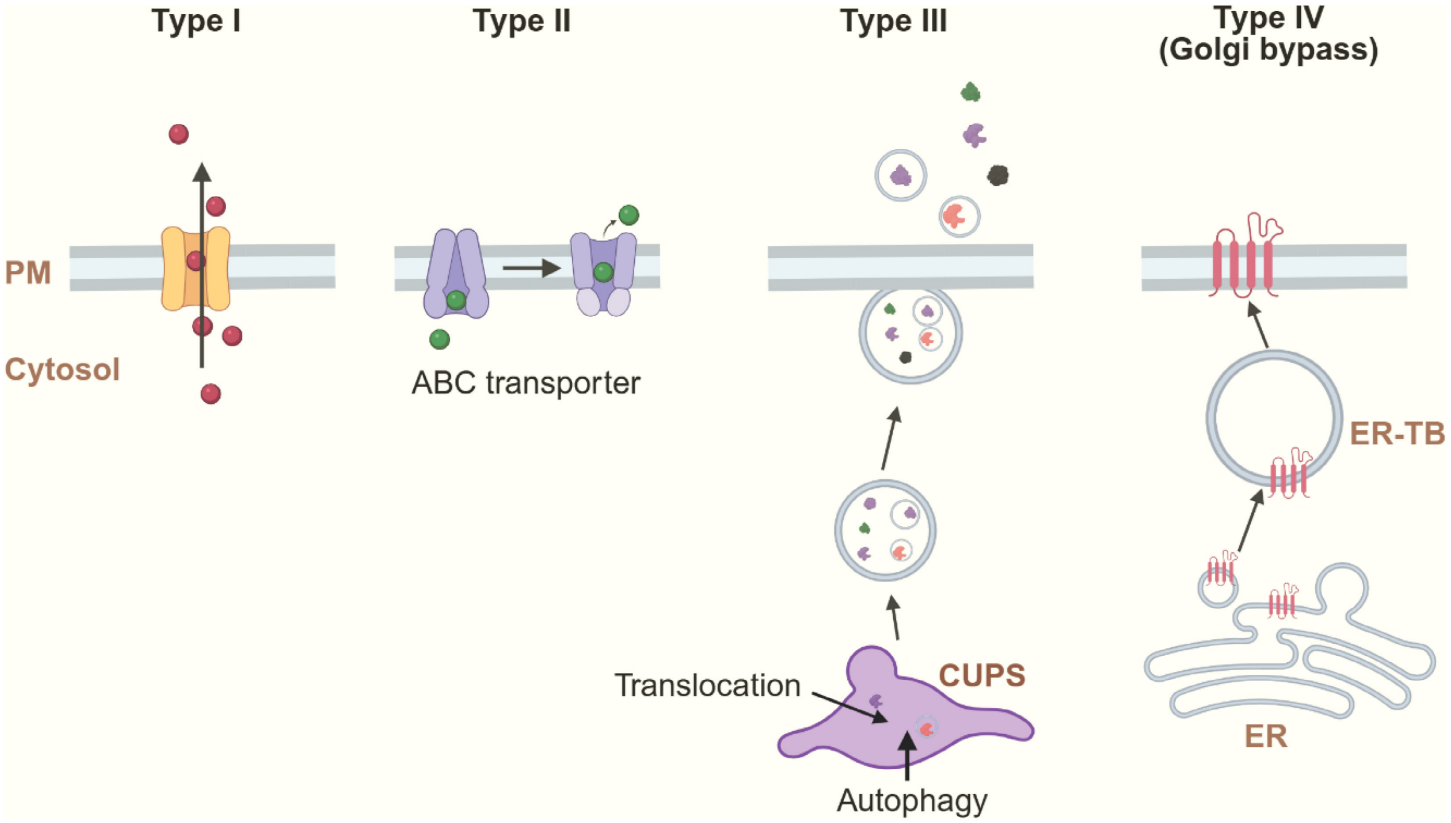
Distinct types of unconventional protein secretion. In type I secretion, cytosolic cargoes are translocated directly across the plasma membrane via a protein or lipid pore. Type II secretion refers specifically to ABC transporter‐mediated secretion, engaging two ATP‐dependent conformational states for cargo binding and release, respectively. Type III secretion requires the entry of cargoes into the lumen of a vesicle‐based secretory compartment or compartment for unconventional protein secretion (CUPS), either by protein translocation or autophagy‐mediated cargo incorporation (see Figure 3). In *S. cerevisiae* , CUPS, formed by Golgi‐derived membranes, appears to interact with the trans‐Golgi network (TGN) to generate vesicles carrying the UcPS cargo Acb1. In type IV secretion, ER membrane proteins are packed into ER‐derived vesicles, which then form a large structure termed ER tubular body (ER‐TB). Cargoes are delivered to the cell surface without traversing through the Golgi apparatus. This figure was generated by BioRender.

The first substrate of UcPS was interleukin‐1β (IL1β), an inflammation‐induced cytokine released by activated macrophages [3]. Since then, the repertoire of UcPS cargos has expanded to include signaling molecules such as Fibroblast Growth Factor 2 (FGF2), Acyl‐CoA binding protein 1 (Acb1), galectins, and engrailed, as well as protein quality control substrates like tau and α‐synuclein (α‐syn) [4]. Multiple mechanisms mediate their secretion (Figure 1): in type I and type II UcPS, cargos translocate directly across the plasma membrane; in type III UcPS, cargos first enter the lumen of an intermediate secretory compartment; and in type IV UcPS, some signal sequence–bearing membrane proteins bypass the Golgi to reach the plasma membrane [5].

The route of secretion determines whether cargos are released as soluble proteins or in association with the plasma membrane or extracellular vesicles (EVs)/exosomes, which in turn influence how these cargos affect target cells. In addition, recent studies have identified tunneling nanotubes (TNTs)—membranous conduits that connect distant cells to transfer proteins or organelles, enabling direct intercellular communication and circumventing extracellular exposure. Together, these diverse mechanisms define the emerging framework of UcPS, a field offering exciting opportunities for discovery. Unsurprisingly, dysregulation of UcPS has been linked to numerous human diseases (see below). This meeting showcases a wide range of UcPS mechanisms, with particular emphasis on types I, III, and IV UcPS.

## 2 The Trafficking Routes and Secretory Compartments in UcPS

**FIGURE 2:**
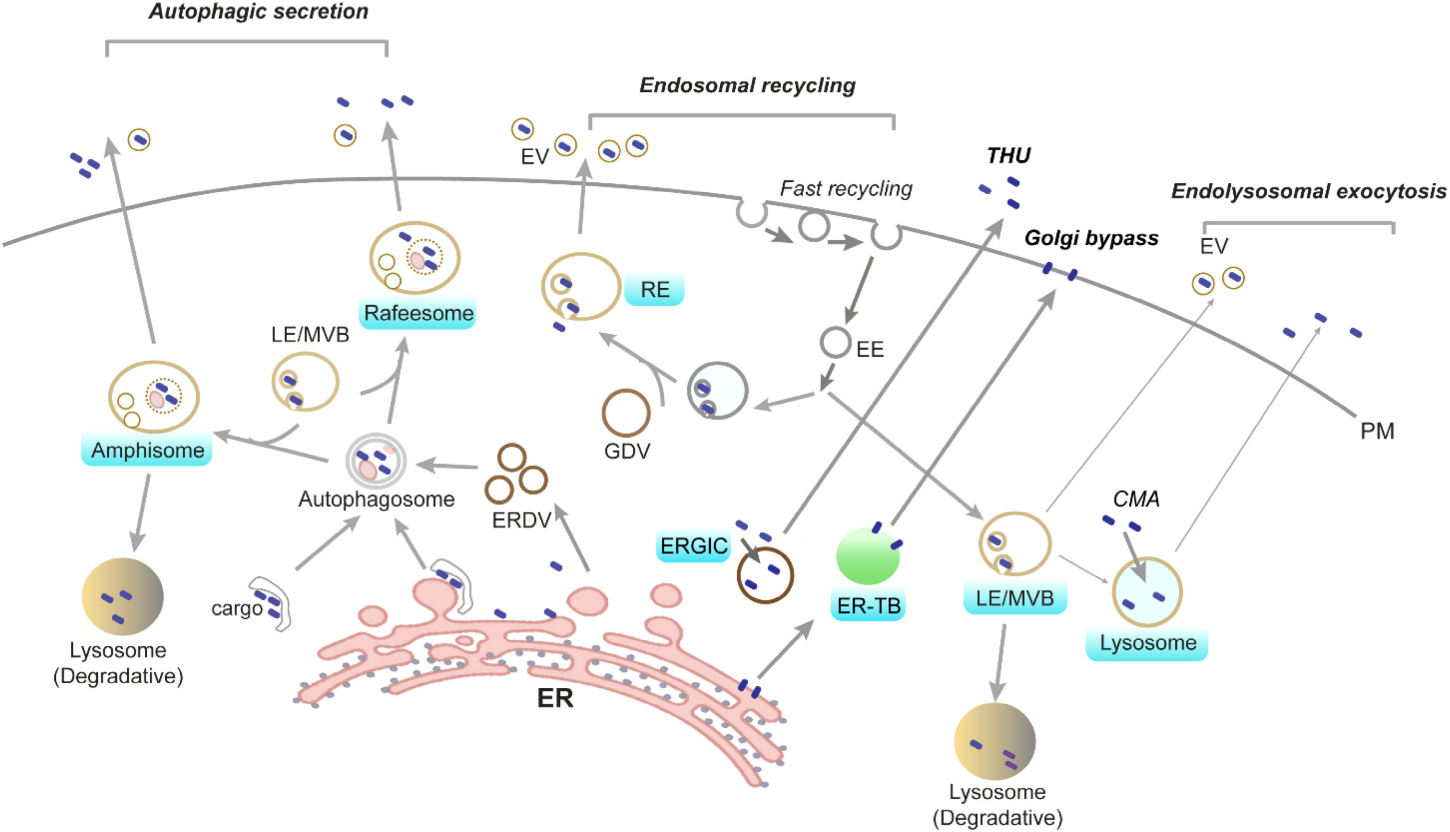
Intermediate compartments in vesicle‐based unconventional protein secretion in mammalian cells. UcPS cargoes can be secreted via a variety of vesicular compartments (indicated by blue labels), provided that they have access to the lumen of these compartments and that these compartments acquire proper targeting and fusion machinery to facilitate the interaction with the plasma membrane (PM). CMA, chaperone‐mediated autophagy; EE, early endosome; ERDV, ER‐derived vesicle; ER‐TB, ER tubular body; EV, extracellular vesicle; GDV, Golgi‐derived vesicle; LE, late endosome; MVB, multivesicular body; RE, recycling endosome; THU, TMED10‐channeled unconventional protein secretion.

The evolution of membrane‐bound organelles in eukaryotic cells has enabled diverse compartmentalized protein secretion pathways, in contrast to prokaryotes, which must export proteins directly across the plasma membrane. In the canonical secretory pathway, newly synthesized secretory and membrane proteins are translocated into the ER or integrated into the ER membrane for folding and assembly [6]. They are then packaged into vesicles bound for the Golgi apparatus en route to their final destinations. With the exception of a few substrates studied to date (e.g., FGF2), many UcPS cargos also use vesicular intermediates in trafficking to the cell surface. These vesicular UcPS pathways, classified as type III and IV, are resistant to Golgi‐disrupting agents such as Brefeldin A. They employ diverse secretory compartments, including ER‐ or Golgi‐derived structures or post‐Golgi vesicles (Figure 2). These compartments act as critical intermediates, enabling distinct protein export routes under stress or pathological conditions when conventional pathways are impaired. Numerous reports at this meeting examined the origin, diversity, and regulation of such compartments in UcPS.

Golgi Reassembly and Stacking Proteins 55 and 65 (GRASP55/65 or GORASPs) are peripheral Golgi/ER proteins implicated in vesicular UcPS. Keynote speaker Vivek Malhotra described how his group established the yeast GRASP homolog Grh1 as a critical regulator of UcPS, dispensable for conventional secretion [7]. In yeast, Acyl‐CoA Binding Protein (Acb1) secretion under carbon and nitrogen starvation requires Grh1 and a specialized compartment, CUPS (Compartment for Unconventional Protein Secretion) [8]. Under nitrogen starvation, Grh1 localizes to CUPS, which interacts with a modified trans‐Golgi network (TGN) that becomes vesiculated during prolonged starvation. Grh1 appears to act in Acb1 delivery to the modified TGN. The ATPase Drs2 flips phosphatidylserine to promote membrane blebbing; Acb1‐containing blebs are severed by ESCRT machinery and fuse with the plasma membrane via Snc1/Snc2 SNAREs [9]. Thus, under starvation, cells assemble CUPS and a modified TGN to export proteins like Acb1 without conventional ER coat proteins.

Antonio Costa‐Filho discussed potential mechanistic roles of GRASP proteins in yeast Acb1 secretion and mammalian UcPS. Under stress‐mimicking conditions, both yeast Grh1 and human GRASP55 formed biomolecular condensates via liquid–liquid phase separation (LLPS) [10]. Notably, Grh1 condensates could recruit Acb1 [11], suggesting that these membrane‐less, liquid‐like assemblies may facilitate selective cargo recruitment to promote UcPS.

Yanzhuang Wang proposed an alternative GRASP‐mediated UcPS mechanism in mammalian cells under metabolic stress. During glucose deprivation, GRASP55 undergoes de‐O‐GlcNAcylation and relocates to autophagosomes, promoting their fusion with lysosomes. This pathway appears to regulate UcPS of aggregation‐prone proteins such as tau and huntingtin through lysosomal exocytosis [12]. He suggested that restoring Golgi integrity and GRASP55 function might enhance lysosomal performance and reduce pathogenic protein accumulation in neurodegenerative diseases.

Several presentations emphasized post‐Golgi compartments as critical intermediates in UcPS, including endosomes (e.g., recycling and late endosomes), autophagosomes, and lysosomes. Thierry Galli reported that late endosome‐associated v‐SNARE VAMP7 mediates the secretion of ER‐ and mitochondria‐derived proteins, exemplified by RTN3A and VDAC, respectively. VAMP7 directs these cargos to late endosomes for extracellular release, bypassing canonical autophagy [13]. Loss of VAMP7 function increased tumor necrosis and growth in a grafted rat glioma model, underscoring its critical role in stress adaptation and organelle crosstalk.

Julien Villeneuve showed that lysosomes can serve as secretory intermediates for UcPS of tau and α‐syn via lysosomal exocytosis. Genetic and proteomic screens identified regulators such as prosaposin (PSAP) that control this process [14, 15]. These results support the concept of a functional lysosomal switch from degradation to secretion, particularly in neurodegenerative contexts [16], although the underlying molecular mechanisms remain unresolved.

Tiebang Kang's group described a novel ER‐derived compartment, the “rafeesome,” a multivesicular body (MVB)‐like structure involved in LC3 and STING secretion [17]. STING, a cytosolic DNA sensor upstream of inflammasome activation, can influence immune signaling in neighboring tumor cells when secreted via this mechanism. UcPS of STING requires ER‐derived vesicles that are generated via TMEM33‐dependent RTN4b oligomerization, which induces high‐curvature membranes to vesiculate the ER. These vesicles fuse with Rab22a‐positive endosomes to form noncanonical autophagosomes—termed rafeesomes [18]—that may be functionally analogous to amphisomes, an autophagosome–endosome fusion product. Both rafeesomes and amphisomes are likely bifunctional, targeting cargos for either secretion or lysosomal degradation depending on cellular contexts (Figure 2).

Autophagy components also contribute to UcPS, primarily through the regulation of secretory autophagy and exosome biogenesis (see below). Min Goo Lee described ER‐tubular bodies (ER‐TBs)—newly identified ER‐derived structures formed under stress conditions such as ER stress and viral infection through the action of the ER autophagy (ER‐phagy) receptors ATL3 and RTN3L [19]. These ER‐TBs mediate the UcPS of transmembrane proteins, including ΔF508‐CFTR and the SARS‐CoV‐2 spike protein, in an autophagy‐dependent but Golgi‐independent manner [20, 21], thereby linking ER‐phagy to Golgi‐bypass secretion during cellular stress. Consistent with Min Goo's presentation, Georgia Maria Sagia's work suggests that type IV UcPS in filamentous fungi *Aspergillus nudulans* involves similar ER‐derived membrane structures. These structures mark a distinct population of ER exit sites that contain UcPs cargo UapA, but not conventional secretory cargos [22].

Vassiliki Nikoletopoulou reported that a subset of LC3‐ and Sec22b‐positive autophagic vesicles participates in the UcPS of synaptic proteins in neurons. Disruption of autophagic vesicle biogenesis impaired the plasma membrane localization of Golgi‐bypass cargos, suggesting a specialized autophagy‐dependent pathway for surface protein delivery in neurons [23]. In addition, several investigators presented the role of autophagy components in extracellular vesicle (EV) secretion via the production of intraluminal vesicles (ILVs) at late or recycling endosomes, which will be discussed in a later section of this report.

Collectively, these presentations highlight the emerging view that UcPS is mediated by a diverse repertoire of stress‐sculpted, non‐canonical secretory compartments—including CUPS, ER‐tubular bodies, rafeesomes, late endosomes, autophagic vesicles, and lysosomes. These compartments are dynamically regulated by a network of molecules such as GRASPs, Rab proteins, SNAREs, and autophagy‐related proteins. Studies on the assembly and function of these context‐specific secretory intermediates not only deepen our understanding of how intracellular trafficking adapts to stress but also reveal new opportunities for therapeutic intervention in diseases characterized by proteostasis imbalance, trafficking defects, and chronic inflammation.

## 3 Substrate Recognition and Membrane Targeting in UcPS

Conventional protein secretion is initiated by the recognition of an N‐terminal signal sequence or a transmembrane domain on a nascent polypeptide as it emerges from the ribosomal exit tunnel. This recognition is mediated by the signal recognition particle (SRP), which directs nascent chains to the heterotrimeric Sec61 complex in the ER membrane. Accordingly, most conventional secretion occurs co‐translationally in a constitutive manner [6]. In contrast, unconventional protein secretion (UcPS) typically occurs post‐translationally—after protein synthesis is complete—and is often triggered by cellular stress. Despite these differences, UcPS likely follows a similar general principle: a signal motif within a cargo is recognized by a cellular factor, which then directs the cargo to a specific secretory intermediate compartment. Given the diversity of membrane compartments involved in UcPS, multiple substrate recognition and targeting mechanisms are likely to exist. Although this topic was not extensively discussed at the meeting, we include a brief summary here for completeness, reflecting our current understanding of cargo recognition in UcPS pathways.

In type I UcPS of FGF2, membrane recruitment of FGF2 is mediated by its specific binding to PI(4,5)P2 in the plasma membrane. This process is facilitated by the Na,K‐ATPase (ATP1A1) and the Tec kinase‐mediated phosphorylation of FGF2, with the former acting as a recruiting factor [24]. Biochemical and structural studies have identified a high affinity and several weaker PI(4,5)P2 binding sites in FGF2 [25, 26]. Additionally, biochemical and structural studies have also revealed how FGF2 is recognized by the α1 subunit of the Na,K‐ATPase [24, 27]. Thus, while the Na,K‐ATPase is believed to act as a landing platform for FGF2 at the inner plasma membrane leaflet, the handover of FGF2 to PI(4,5)P_2_ triggers FGF2 membrane translocation and secretion.

**FIGURE 3:**
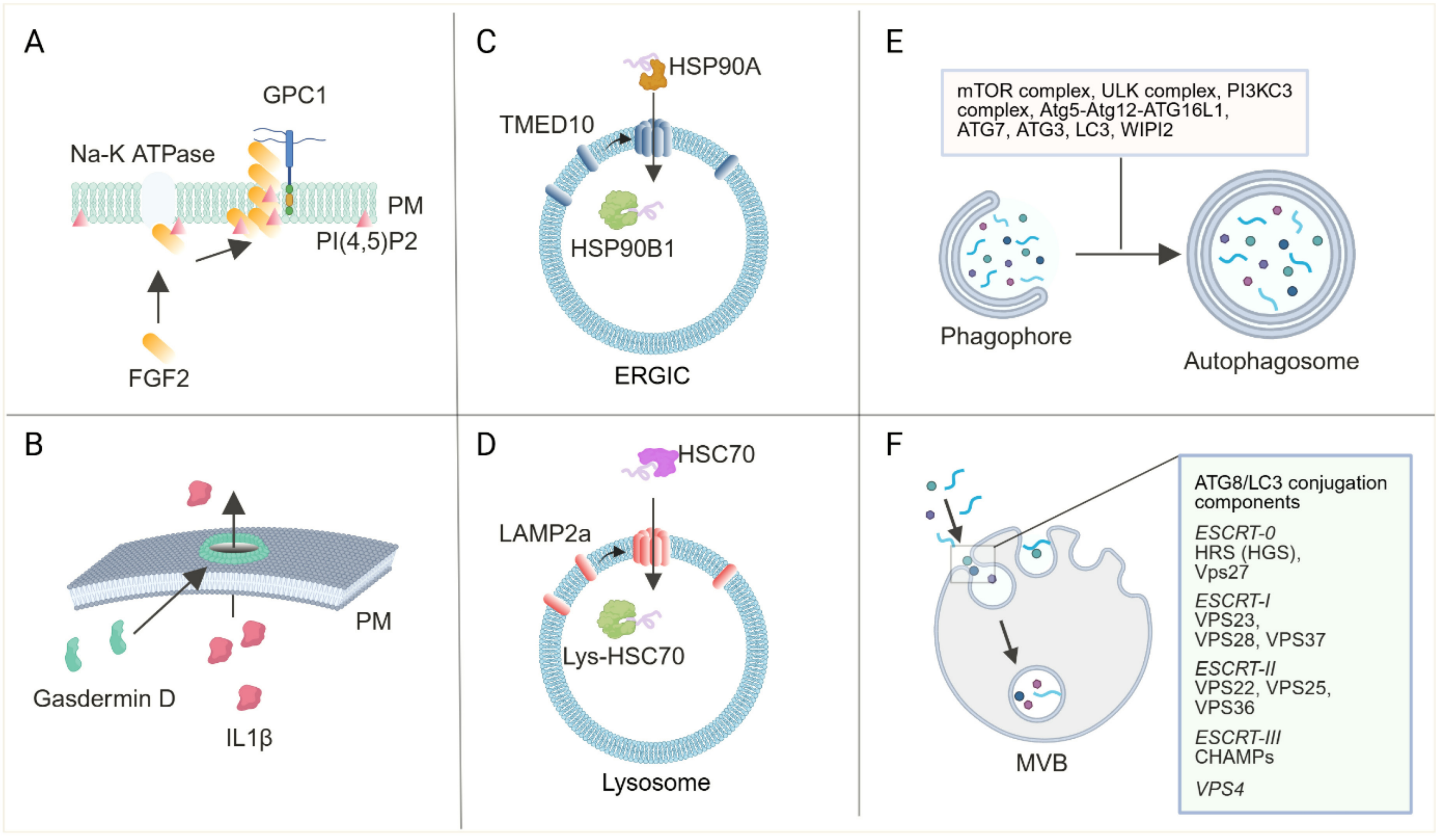
Distinct protein translocation mechanisms in unconventional protein secretion. (A) FGF2 translocation across the plasma membrane by self‐assembled lipid pore. The process requires Na‐K ATPase for efficient FGF2 membrane recruitment and the asymmetric distribution of PI(4,5)P2 for pore formation. GPC1 grabs emerging FGF2 in the extracellular space to prevent cargo back‐sliding. PM, plasma membrane. (B) Inflammasome activation induces the self‐assembly of Gasdermin D into a membrane pore, allowing IL1β to diffuse across the plasma membrane together with other cytosolic contents. (C) In TEME10‐channeled UcPS, cytosolic HSP90A chaperones cargoes to an ERGIC compartment. Cargoes are unfolded and translocated into the lumen. This process is facilitated by homo‐oligomerization of TMED10 and a luminal HSP90 variant, HSP90B1. (D) In chaperone‐mediated autophagy, cytosolic cargoes are recognized by HSC70, and then translocated into the lysosomal lumen via a homo‐oligomerized LAMP2a structure and a lysosomal (lys‐) HSC70 variant. (E) Macroautophagy incorporates cytosolic cargoes into the lumen of autophagosomes. Cargoes are surrounded by double membranes. The box indicates some key autophagic regulators. (F) Components of the ATG8/LC3 conjugation and microautophagy machinery incorporate cytosolic cargoes after they are targeted to the surface of late endosomes. Assisted by the ESCRT complexes (listed in the box), membranes of late endosomes invaginate and bud into the lumen, generating a multivesicular structure that contains cytosolic cargoes with intraluminal vesicles. This figure was generated by BioRender.

Given the stress‐regulated nature of many of the UcPS mechanisms, substrate recognition in these pathways is likely tightly linked to cytosolic protein quality control mechanisms involving molecular chaperones. Consistent with this view, GRASP55 was suggested to possess an intrinsic chaperone activity that prevents IL1β aggregation in UcPS [28]. In type III secretion of IL1β, caspase cleavage appears to expose two sequence motifs that are recognized by the cytosolic chaperone HSP90. The HSP90–IL1β complex is subsequently targeted to the ER–Golgi intermediate compartment (ERGIC), likely through a direct or indirect interaction with the cargo receptor TMED10 [29]. Under conditions of proteasome deficiency, cells activate a distinct protein quality control pathway known as Misfolding‐Associated Protein Secretion (MAPS). This process is initiated by USP19, an ER‐associated deubiquitinase with intrinsic chaperone activity, which also interacts with HSP90 and HSC70 [30]. Together with HSC70 and the DnaJ family co‐chaperone DNAJC5, USP19 promotes the recruitment of misfolded proteins to the ER surface, where they are targeted for secretion via a Golgi‐derived compartment or late endosomes [31, 32, 33]. This trafficking step may be facilitated by ER–late endosome contact sites, which would enable proximity‐based cargo transfer. Finally, a related HSC70‐dependent quality control mechanism—chaperone‐assisted autophagy (CMA)—targets aberrant cytosolic proteins to lysosomes for degradation via a putative protein translocation channel formed by LAMP2a (Figure 3) [34]. The primary function of translocating aberrant cytosolic proteins to membrane‐bound compartments is presumed to target them for lysosomal degradation. However, under stress conditions, these compartments, including lysosomes themselves, can acquire the capacity to fuse with the plasma membrane, thereby activating distinct UcPS routes. Consistent with this view, a recent study showed that lysosomal damage triggers lysosomal exocytosis [35].

Cargo recognition and targeting to intraluminal vesicles (ILVs) during EV biogenesis is likely coordinated with distinct forms of selective autophagy. In a lysosome‐mediated secretion pathway named LC3‐Dependent EV Loading and Secretion (LDELS) (see below), RNA‐binding proteins are directed to LC3‐positive late endosomes through LC3‐interacting region (LIR) motifs within cargos [36]. Their incorporation into ILVs is presumed to occur via ESCRT‐dependent mechanisms similar to those utilized for microautophagy.

In another example of ILV‐mediated secretion, the β‐galactoside‐binding lectin galectin‐3 (Gal3) is directly recognized by the ESCRT‐I component Tsg101 through a conserved P(S/T)AP ‘late domain’ motif located in its N‐terminal region [37]. Targeted knockdown of Tsg101 or mutation of this motif blocks Gal3 incorporation into EVs, as presented by Rafe Jacob. This late domain‐mediated sorting mechanism may also facilitate the inclusion of other proteins—such as E‐cadherin—into EVs [38], supported by bioinformatic analyses that have identified hundreds of proteins containing this motif. Additionally, the frequent detection of RTN3—an ER‐resident macroautophagy receptor—in secreted EVs raises the possibility that macroautophagy, or ERphagy in particular, might contribute to cargo recognition and selection in UcPS [13, 23]. Together, these findings suggest that multiple autophagy‐related pathways may intersect with EV biogenesis to mediate selective cargo incorporation.

## 4 Protein Translocation Across Membranes in UcPS

A central question in protein secretion is how cargos are translocated across at least one membrane, given that the cytoplasm is separated from the cell exterior—which is topologically equivalent to the lumen of organelles such as the ER and Golgi—by a lipid bilayer. In conventional secretion, nascent polypeptides are translocated into the ER lumen via the conserved Sec61 translocon, a heterotrimeric protein‐conducting channel. This process has been reconstituted in vitro with purified components, and recent cryo‐EM studies have shed light on how cargos engage the translocon to activate the translocation process [6]. By contrast, protein translocation mechanisms in UcPS are more diverse and far less understood.

The best‐characterized UcPS translocation pathway is the secretion of FGF2 via type I UcPS in mammalian cells (Figure 3). Following its recruitment to the inner plasma membrane leaflet by the Na,K‐ATPase, PI(4,5)P2 binding triggers FGF2 oligomerization, driving the formation of a transient toroidal lipid pore. Membrane‐proximal heparan sulfate chains of Glypican‐1 (GPC1) capture FGF2 oligomers within the pore, enabling directional translocation to the cell surface [39]. PI(4,5)P2, a negatively charged lipid confined to the inner leaflet, accumulates locally during FGF2 oligomerization, generating a steep, spatially restricted electrochemical gradient. At high local concentrations, PI(4,5)P2's wedge‐like geometry destabilizes the bilayer, favoring pore formation [26]. Walter Nickel and colleagues showed that asymmetric PI(4,5)P2 distribution across the bilayer accelerates FGF2 pore opening, and disrupting this asymmetry inhibits FGF2 secretion [40]. They propose that PI(4,5)P2 asymmetry lowers the energetic barrier for pore formation to facilitate rapid FGF2 translocation—a principle potentially applicable to other type I UcPS cargos whose translocation involves transient membrane pores and cell‐surface heparan sulfates acting as an extracellular trap [41, 42]. This model is further supported by a molecular simulation study by Fabio Lolicato.

Another type I UcPS translocation mechanism involves Gasdermin‐generated plasma membrane pores (Figure 3), which, if not repaired immediately, drive pyroptosis and the release of cytoplasmic proteins such as IL1β and other cytokines from macrophages, epithelial, and endothelial cells. Pyroptosis, a lytic form of cell death marked by swelling and membrane rupture, was first described upon inflammasome activation in macrophages during bacterial invasion. Mammalian innate immunity uses canonical and noncanonical inflammasomes, both activating Gasdermin D. In the canonical pathway, caspase‐1 cleaves Gasdermin D, whose N‐terminal domain oligomerizes and inserts into the membrane, forming ~180 Å pores essential for IL1β secretion and autoinflammatory pathology in Pyrin‐ or NLRP3‐driven mouse models [43]. In the noncanonical pathway, intracellular LPS activates caspase‐4/5 in epithelial cells, triggering Gasdermin D–dependent IL18 secretion [44]. Recent work from Feng Shao's group revealed that caspase‐4—but not caspase‐11—forms a specific complex with pro‐IL18 to activate it. Mouse studies further showed that caspase‐4‐dependent activation of Gasdermin D in brain endothelial cells contributes to bacterial‐ and LPS‐induced blood–brain barrier (BBB) damage, identifying a potential therapeutic target for preserving BBB integrity during infection or sepsis [45]. Shao's group also reported Gasdermin functions in ancient eukaryotes, including fungi and *Trichoplax adhaerens* [46], establishing Gasdermins as an evolutionarily conserved secretory machinery.

While IL1β release from macrophages in mice is primarily mediated by Gasdermin‐assembled plasma membrane pores, Liang Ge reported a type III UcPS pathway that exports IL1β and many cytosolic proteins from epithelial cells in vitro. This pathway also appears to contribute to IL1β release in cecal ligation puncture‐treated mice. In this secretion process, IL1β is first translocated into the ER‐Golgi intermediate compartment (ERGIC). Membrane translocation requires oligomerization of TMED10 (Tmp21) [29], a single‐pass membrane protein in a family known for cargo sorting at the ER in conventional secretion. While cargo sorting by TMED10 depends on its hetero‐assembly with other TMED family members, Liang's work suggests that protein translocation is mediated by TMED10 homo‐oligomers (unpublished result). Therefore, the pathway was named TMED10‐channeled UcPS (THU) (Figure 3). More recently, Liang found that TMED family members, except TMED6, all possess similar protein translocation activity. They function in the ERGIC compartment, use their cytoplasmic tails for cargo selectivity, and homo‐oligomerize to mediate protein translocation [47]. Furthermore, Ming Zhang identified two small RAB GTPases—Rab1 and Rab2—as key regulators of THU. Rab1 activation promotes TMED10 homo‐oligomerization, while Rab2 regulates cargo sorting at the ERGIC for efficient secretion [48]. Because TMED10's transmembrane domain lacks a typical amphipathic helical structure, homo‐oligomerization alone may not form a protein‐conducting channel. While the mechanism underlying TMED10‐mediated membrane translocation and substrates of THU are being worked out, Juan Wang's presentation linked TMED10 to a non‐canonical autophagy pathway for galectin‐9 secretion, requiring TMED10 and ATG9A‐positive vesicles. While ATG9A is a core autophagy factor, here it functions as a carrier for galectin‐9, with TMED10 enabling its incorporation into ATG9A vesicles. Fusion of these vesicles with the plasma membrane via the STX13–SNAP23–VAMP3 SNARE complex releases galectin‐9 [49]. These findings suggest a potential cooperation between TMED10 and autophagy machinery in protein transport across membranes in UcPS.

In addition to direct membrane translocation, cytosolic UcPS cargos can be incorporated into secretory intermediates via components of the macroautophagy or microautophagy machinery (Figure 3). These pathways use distinct membrane domains to engulf selected cytosolic proteins into vesicles within autophagosomes or endosomes. Normally destined for lysosomal degradation, under stress, these vesicles can instead fuse with the plasma membrane, releasing their contents along with intraluminal vesicles (ILVs). This stress‐induced rerouting is a major source of EVs. Although autophagic targeting of cytosolic cargos was not a focus of this meeting, the EV biogenesis session highlighted how cells package cytosolic proteins into ILVs in endosomes and redirect these vesicles from degradation toward secretion (see below).

## 5 Extracellular Vesicle Biogenesis and Secretion

Intraluminal vesicle (ILV)‐containing late endosomes (also termed multivesicular bodies, MVBs) are considered major precursors of exosomes or endosome‐derived EVs. Jay Debnath reported two autophagy‐related unconventional protein secretion (UcPS) pathways—SALI (Secretory Autophagy during Lysosome Inhibition) [50] and LDELS [36]—that contribute to ILV biogenesis. SALI is a regulated secretory process that promotes EV release upon lysosomal inhibition, whereas LDELS constitutively mediates the secretion of small EVs formed at late endosomes during MVB biogenesis. Both pathways require ATG7, ATG12, and Rab GTPase Rab27a, but SALI uniquely depends on additional autophagic regulators such as ATG14, ATG2, and FIP200 that are dispensable for LDELS. Instead, LDELS involves the conjugation of the ubiquitin‐like molecule ATG8/LC3 to single membranes, a process known as CASM (Conjugation of ATG8 to Single Membranes) [51]. LDELS cargos are predominantly RNA‐binding proteins that contain LC3‐interacting region (LIR) motifs, enabling their recruitment to LC3‐decorated limiting membranes and subsequent incorporation into ILVs. Inhibition of the LDELS pathway through siRNA‐mediated knockdown led to the accumulation of RNA granules and disruption of RNA metabolism, suggesting a key role for LDELS in maintaining RNA homeostasis.

The fusion of MVBs with the plasma membrane instead of lysosomes releases ILVs as exosomes. However, the mechanisms specifying the fate of MVBs remain poorly understood. To address this question, Frederik Verweij's group developed an optical reporter, CD63‐pHluorin, which enables real‐time visualization of MVB–plasma membrane fusion at the single‐cell level using live TIRF microscopy [52]. Using this system, they demonstrated that cells release exosomes at low levels under steady‐state conditions, but extracellular stimuli—such as histamine—can enhance MVB–plasma membrane fusion. Histamine treatment activates PKCα, leading to the phosphorylation of the SNARE protein SNAP23, thereby promoting fusion. This secretory mechanism also requires Rab27a, while MVB–lysosome fusion is governed by a distinct RAB GTPase, Rab2a. Interestingly, Rab2a loading appears to occur at the ER–MVB contact sites, which in turn exclude Rab27a association [53]. ER–MVB contact sites likely play multiple roles in UcPS, suggested by Jay Debnath. Their study highlighted a role for the ER–late endosome contact sites in ceramide transport from the ER to MVBs via the FAN/SMPD3 complex located at these interfaces. Ceramide transport enhances LDELS‐mediated ILV release, but the underlying mechanism is unclear (unpublished results). The conversion of degradation‐bound late endosomes into a secretory intermediate may involve additional lipid—particularly phosphatidylinositol 4‐phosphate (PI4P). Supporting this idea, recent work from Wei Guo's group showed that the activation of a PI4P kinase on MVBs leads to PI4P accumulation, which in turn recruits effectors such as the exocyst complex. These effectors tether MVBs to the plasma membrane, promoting membrane fusion and cargo release [54]. PI4P may also participate directly in the formation of ILVs, as suggested by Yin Hang [55].

In addition to late endosomes, recycling endosomes—originating from a subset of early endosomes—can also contribute to EV production. This pathway specifically generates Rab11a‐positive EVs, as reported by Deborah Goberdhan [56]. In collaboration with Clive Wilson (also at Oxford), Deborah developed a *Drosophila* model to study EV production in prostate‐like secondary cells, which contain enlarged Rab11a‐positive recycling endosomes bearing intraluminal vesicles (ILVs). Combining proteomics with a gene knockdown approach, they identified several conserved accessory ESCRT‐III proteins that function as both EV cargoes and selective regulators of Rab11a‐mediated EV production [57]. These Rab11a‐positive ILVs appear to form when E‐cadherin‐enriched lipid platforms invaginate from the limiting membrane in a manner analogous to ESCRT‐mediated MVB biogenesis in late endosomes. The ILV‐containing recycling endosomes can fuse with larger, Golgi‐derived, Rab6‐positive secretory compartments through an ARF1‐dependent mechanism, generating hybrid Rab11a‐positive compartments that contain both secretory dense cores and ILVs [58]. This fusion enables recycling endosomes to subsequently fuse with the plasma membrane, releasing both conventional secretory cargoes and EVs [59]. Furthermore, Rab11a‐positive ILVs are linked to protein aggregation during dense core formation, via proteins and mechanisms involved in Alzheimer's disease.

When and how are early endosomes converted into recycling endosomes? Kangmin He's presentation described a rapid endosomal recycling mechanism known as Clathrin‐Associated fast endosomal Recycling Pathway (CARP) [60]. This pathway involves a population of AP1‐positive, Clathrin‐associated vesicles—termed CARP carriers—that can transiently fuse with the plasma membrane shortly after Clathrin‐mediated endocytosis. Using a specific PI(4,5)P_2_ sensor [61] and advanced imaging techniques, their study demonstrated that CARP carriers undergo ‘kiss‐and‐run’ membrane fusion and lack association with known endosomal retrieval complexes [60]. Instead, these vesicles dynamically recruit a distinct set of trafficking molecules—including Arf1, AP1, Rab1, Rab11, components of the exocyst complex, SNARE proteins, and dynamin2. These molecules may determine the trafficking fate of CARP carriers.

Another molecule implicated in the cell surface delivery of recycling endosomal contents is the nonaspanin protein NSP‐3/TM9SF3, reported by the Zhou and Bessereau labs. Using *C. elegans* as a model, Xin Zhou showed that NSP‐3 is partially localized on recycling endosomes, overlapping with RAB11. Genetic analyses showed that the loss of NSP‐3, or retrograde trafficking components such as SNX‐3 and SNX‐17, disrupts specifically the cell surface recycling of the GABA_A_ receptors, leading to their accumulation and aggregation within early and recycling endosomes. Notably, this phenotype is modulated by mutations affecting proteins involved in the trafficking of Golgi‐derived vesicles, including adaptor complexes AP1–3, the vesicular sorting regulator RME‐8, and lysosomal degradation regulators CUP‐5 and LMP‐1 (unpublished results). These findings place NSP‐3 at a critical intersection between Golgi‐associated secretory fate determination and endosomal cargo sorting, further emphasizing the importance of fusion events between recycling endosomes and Golgi‐derived vesicles in this recycling pathway. Although it remains unclear whether recycling endosomes lacking intraluminal vesicles (ILVs)—such as CARP carriers—can participate in UcPS of soluble cargoes, Shin Hye Noh reported that these compartments can mediate type IV secretion of ER‐derived transmembrane cargos via the retromer complex. Mechanistically, recycling endosomes with or without ILVs may use a common strategy to fuse with the plasma membrane, regardless of whether they carry UcPS cargos.

Anbing Shi's presentation further extended the discussion on the role of recycling endosomes in protein secretion. His talk focused on the role of the small GTPase Rab10 and its effector EHBP‐1 in the basolateral exocytosis of signal sequence‐bearing lipoprotein in the intestinal epithelia of *C. elegans* . EHBP‐1, as a Rab10 effector positioned on recycling endosomes [62], acts as a tether to capture Rab10‐positive vesicles via its coiled‐coil and PI(4,5)P2‐binding C2 domains, thereby facilitating cargo transport to recycling endosomes. This process uses LST‐6/DENND5 but not DENND4 as a specific guanine nucleotide exchange factor (GEF) for Rab10 [63, 64]. This study underscores an unconventional role for recycling endosomes in a conventional protein secretion pathway, illustrating additional cross talk between the two protein secretion processes.

## 6 TNT, Cytoneme and Migrasome: Unconventional Paradigms of UcPS

Alongside EV‐mediated cell–cell communications, cells also employ membrane protrusions for direct or indirect exchange of cytosolic contents. A notable example is migrasomes—vesicular structures that form along retraction fibers during cell migration [65]. First reported by keynote speaker Li Yu and his colleagues, migrasomes remain tethered to migrating cells before detaching to become specialized EVs enriched in tetraspanin microdomains [66] and filled with signaling molecules [67]. These vesicles can be taken up by neighboring cells, delivering bioactive cargos or disposing of damaged organelles [68]. Li Yu's recent work highlighted migrasomes' pivotal role in spatiotemporal control of signaling gradients during animal development and immune responses [69, 70].

Continuing the theme of protrusion‐based communication, Stacey Ogden presented her work on cytonemes—slender (< 200 nm), actin‐based, close‐ended extensions that function as signaling hubs [71]. Cytonemes mediate the targeted transport of morphogens such as Sonic Hedgehog (SHH), Wnt, Notch, BMP, and various growth factors [72], and their formation is regulated by morphogen activity itself [73]. Using the SHH pathway as a model, Ogden's team showed that the transmembrane receptors CDON and BOC are essential for cytoneme formation, while the actin motor MYO10 drives their elongation in SHH‐expressing cells of the developing mouse neural tube [74]. Ongoing studies are to dissect how CDON/BOC receptor activity interfaces with MYO10‐mediated actin dynamics to control cytoneme‐mediated signaling.

A third protrusion‐based structure, tunneling nanotubes (TNTs), is an open‐ended, F‐actin–based cytoplasmic bridge that connects cells across distances of tens to hundreds of microns [75]. Chiara Zurzolo's group has demonstrated TNT‐like structures in vivo that are distinct from cytonemes and cytokinetic bridges. In the developing mouse cerebellum, they connect granule neuronal precursors in their migratory state, suggesting that they may precede synapse formation and facilitate neural network maturation [76]. Her team also reported functional TNT‐like structures in live zebrafish embryos, capable of transferring cytoplasmic material and sensitive to Eps8 overexpression, providing the first direct evidence of functional TNTs in a vertebrate embryo [77]. In adult tissues, TNTs induced under stress or inflammation can be exploited by pathogens such as HIV‐1 and SARS‐CoV‐2 to evade immune detection [78, 79]. In Parkinson's disease models, TNTs facilitate bidirectional exchange between neurons and microglia: α‐syn aggregates are transported from neurons to microglia, while healthy mitochondria move in the reverse direction [80, 81]. Using advanced microscopy in human cell lines and iPSC‐derived neuron–microglia co‐cultures, Zurzolo's group identified defective autophagy as a driver of this directional transfer (unpublished results), suggesting that TNTs may mediate neuroprotective clearance of toxic protein aggregates by delivering them to microglia.

Building on the theme of TNT‐mediated communication, Christel Vérollet presented compelling work on TNT's role in viral spreading and cell fusion. Her team identified Siglec‐1, a lectin involved in macrophage immune signaling, as an essential regulator of TNT‐mediated cell fusion and HIV‐1 spread [82], which is regulated by glycolysis. More recently, they uncovered a new role for TNTs in osteoclast fusion where the ERM family protein moesin functions as a negative regulator [83]. This work broadens the scope of TNT biology by demonstrating their role in fusion‐driven processes, highlighting how structurally distinct TNTs enable diverse functions across cell types and disease contexts.

## 7 Physiological Relevance and New Frontiers in UcPS

The diverse cargoes released by UcPS can execute essential autocrine, paracrine, and endocrine functions across various physiological and pathological contexts, including cellular homeostasis, immune responses, intercellular communication, and disease progression, as highlighted by several presentations at this conference.

One key role of UcPS is delivering bioactive molecules to the cell surface to ensure effective cell–cell communication. Lian Li reported a collection of glycosylated proteins as novel UcPS cargoes. Protein N‐glycosylation begins in the ER lumen, where a 14‐sugar precursor is attached to asparagine residues of nascent chains and is subsequently remodeled in the Golgi to generate diverse glycan structures [84]. Using quantitative glycoproteomics combined with large‐scale N‐glycoform and site‐specific glycan analyses [85], Li showed that oligomannose modification is the predominant N‐glycosylation type in the brain, enriched in neuronal and synaptic membrane proteins. Remarkably, neurons may use the Golgi bypass pathway to deliver some oligomannosylated proteins to the cell surface, thereby maintaining brain homeostasis. Lian suggested that disruption of this pathway and altered N‐glycosylation could cause synaptic dysfunction and contribute to Alzheimer's disease.

Beyond glycoproteins, Jun Lu detected stable glyco‐RNAs on neutrophil surfaces using a clickable sialic acid tracer and BrdU labeling, confirming earlier findings [86, 87]. Surface glyco‐RNAs selectively bind P‐selectin, promoting neutrophil–epithelial adhesion during inflammation. Neutrophils stripped of surface RNAs by RNase treatment failed to migrate to inflammatory sites in mice, demonstrating their essential function in neutrophil maturation and activation [88]. Jun proposed the involvement of an unidentified membrane RNA transporter in surface glyco‐RNA delivery, warranting further investigation.

In addition to surface display, UcPS‐derived EVs act as potent intercellular messengers. Clotilde Thery reported that triple‐negative breast cancer (TNBC) cells release EVs coated with macrophage colony‐stimulating factor 1 (CSF1), which promotes monocyte differentiation into a distinct pro‐inflammatory macrophage subtype. In TNBC patients, these EVs correlate with improved survival and potentially anti‐tumoral immune infiltrate [89]. TNBC cells express both short (S‐CSF1) and long (L‐CSF1) isoforms; each contains a transmembrane domain but differs in glycosylation and cleavage patterns [90]. On EVs, S‐CSF1 is membrane‐anchored via its transmembrane domain, whereas L‐CSF1 is associated with membranes in a cleaved form. EVs bearing either isoform can enhance monocyte survival more effectively than soluble CSF1. However, when inoculated in immunocompetent mice, these EVs differentially influence tumor growth. These findings highlight potential therapeutic applications of CSF1‐bearing EVs in anti‐tumor immunotherapy.

Along the theme of EV being a functional messenger, Ying Zhang showed that EVs from activated macrophages deliver both TLR9‐stimulating DNA, such as cytosine‐guanine oligodeoxynucleotide (CpG ODN), and the protein Cdc42 into target cells [91]. This dual transfer triggers immune activation and promotes further EV uptake, amplifying innate immunity. Hang Yin also reported EVs as key mediators of non‐canonical secretion for several innate immune regulators. Specifically, they showed that the ESCRT‐0 adaptor STAM promotes the packaging of activated STING oligomers into EVs, thereby attenuating STING‐driven immune responses [92]. Other functional EV cargoes discussed include PTEN, the oncogenic EGFRvIII mutant, and PD‐L1, which drive intercellular communication favoring cancer progression [93]. Additionally, EVs can also mediate the secretion of mRNAs. In this regard, Ying Zhang reported the identification of several RNA‐binding proteins that selectively package mRNAs into EVs through RNA‐binding motif–driven phase separation, providing a mechanism for mRNA sorting (Unpublished results).

UcPS also contributes to protein homeostasis, and its dysregulation has been linked to age‐related neurodegeneration. In Parkinson's disease, propagation of misfolded α‐syn likely involves UcPS‐mediated release and uptake by neighboring cells [94, 95]. Shenjie Wu demonstrated that the palmitoylation of chaperone DNAJC5 is essential for α‐syn secretion, independent of α‐syn oligomerization or folding state [31]. Wu further identified membrane protein candidates potentially mediating α‐syn membrane translocation during secretion (unpublished result). Independently, Yihong Ye confirmed DNAJC5's role, placing it downstream of USP19 in chaperoning α‐syn to the extracellular space [32]. Palmitoylation‐deficient DNAJC5 mutants are defective in MAPS [31, 96]; they form aggregates and damage lysosomal membranes, leading to neurodegeneration [97]. These results suggest that while UcPS‐mediated clearance of misfolded proteins may protect individual cells, it can harm neighboring cells if released aggregates are not promptly removed.

Finally, UcPS pathways can be hijacked by viruses to traffic viral proteins or modulate host immunity. As mentioned above, type IV UcPS delivers SARS‐CoV‐2 Spike protein to the cell surface [19], which might facilitate Spike‐mediated cell–cell fusion and severe COVID‐19 [98]. Min Zhang showed that envelope (E) proteins from SARS‐CoV‐2, SARS‐CoV, and MERS‐CoV trigger IL1β release via TMED10‐chaperoned UcPS, exacerbating lung inflammation. These proteins promote TMED10 oligomerization to facilitate cargo translocation into the ERGIC [99], revealing TMED10 as a potential therapeutic target for treating severe COVID‐19.

## 8 Perspectives and Future Directions

Over the last few decades, the study of UcPS has evolved from cataloging distinct pathways—such as type I–IV secretions, EV release, migrasome formation, cytoneme‐mediated signaling, and tunneling nanotube (TNT) transport—to exploring the molecular mechanisms as well as the physiological roles of UcPS in animal development, stress adaptation, and disease progression. Despite intensive studies, many fundamental questions remain unresolved. How do cells coordinate the selection and targeting of cargos among distinct UcPS pathways, and to what extent are these mechanisms redundant or specialized? What types of targeting signals are there for cargo selection and how are they recognized in the cell? How are cargo proteins translocated or incorporated into a secretory membrane compartment? What molecular switches determine whether these membrane compartments direct cargos to lysosomal degradation versus secretion? How are the protrusion‐ or vesicle‐based routes integrated with canonical trafficking systems and autophagy, particularly under physio‐pathological stress? Does UcPS play a role in shaping the tumor microenvironment and can UcPS cargos be used as biomarkers for disease diagnosis or therapeutic evaluation? Advancing the field will require in vivo imaging at high spatiotemporal resolution, systems‐level proteomics to define pathway‐ or disease‐specific cargos, and functional perturbations in physiological contexts ranging from development to neurodegeneration. Ultimately, a mechanistic understanding of UcPS could open new therapeutic avenues—either by blocking pathogenic protein spread or by harnessing these pathways for targeted delivery of therapeutic cargos and for disease biomarker development.

## Author Contributions

All authors contributed a summary of their presentation. Session chairs (W.N., M.G.L., Y.Y., M.Z., and C.Z.) assembled the summaries for their session. Y.Y. revised the text and generated the figures. All authors proofread the manuscript. Authors are listed alphabetically.

## Conflicts of Interest

The authors declare no conflicts of interest.

## Peer Review

The peer review history for this article is available at https://www.webofscience.com/api/gateway/wos/peer‐review/10.1111/tra.70022.
